# Frequency of use of the religious exemption in New Jersey cases of determination of brain death

**DOI:** 10.1186/s12910-018-0315-0

**Published:** 2018-08-14

**Authors:** Rachel Grace Son, Susan M. Setta

**Affiliations:** Northeastern University, 371 Holmes Hall, 360 Huntington Ave, Boston, MA 02115 USA

**Keywords:** Religious exemption, Frequency, Brain death, New Jersey, Medical ethics, Determination of death, Neurological criteria of death, Orthodox Judaism

## Abstract

**Background:**

The 1981 Uniform Determination of Death Act (UDDA) established the validity of both cardio-respiratory and neurological criteria of death. However, many religious traditions including most forms of Haredi Judaism (ultra-orthodox) and many varieties of Buddhism strongly disagree with death by neurological criteria (DNC). Only one state in the U.S., New Jersey, allows for both religious exemptions to DNC and provides continuation of health insurance coverage when an exception is invoked in its 1991 Declaration of Death Act (NJDDA). There is yet no quantitative or qualitative data on the frequencies of religious exemptions in New Jersey. This study gathered information about the frequency of religious exemptions and policy in New Jersey that was created out of respect for religious beliefs.

**Methods:**

Literature and internet searches on topics related to religious objections to DNC were conducted. Fifty-three chaplains and heads of bioethics committees in New Jersey hospitals were contacted by phone or email requesting a research interview. Respondents answered a set of questions about religious exemptions to DNC at the hospital where they worked that explored the frequency of such religious exemptions in the past five years, the religious tradition indicated, and whether any request for a religious exemption had been denied. This study was approved by the Northeastern University Institutional Review Board (IRB #: 16–03-15).

**Results:**

Eighteen chaplains and bioethics committee members participated in a full research interview. Of these, five reported instances of religious exemptions to DNC occurring at the hospital at which they worked for a total of approximately 30–36 known exemptions in the past five years. Families sought religious exemptions because of faith in an Orthodox Judaism tradition and nonreligious reasons. No failed attempts to obtain an exemption were reported.

**Conclusions:**

Religious exemptions to DNC in New Jersey do occur, although very infrequently. Prior to this study, there was no information on their frequency. Considering religious exemptions do occur, there is a need for national or state policies that addresses both religious objections to DNC and hospital resources. More information is needed to better understand the impact of granting religious exemptions before new policy can be established.

## Background

The freedom to practice religious beliefs is a fundamental right in the United States. Unfortunately, freedom of religion can conflict with accepted medical standards. The Uniform Determination of Death Act (UDDA) established that either cardio-respiratory or neurological criteria of death are both valid in 1981 [1]. Although most forms of Christianity as well as some varieties of Judaism and Islam find these criteria appropriate, other religious traditions including most forms of both Haredi Judaism and varieties of Buddhism strongly disagree with determining death by using neurological criteria (DNC) [2].

Only one state in the United States, New Jersey, requires use of the cardio-respiratory definition of death and provides for continuation of health insurance coverage when there is a religious exemption to DNC. This comes through the 1991 New Jersey Declaration of Death Act (NJDDA) [3]. Prior to the adoption of the NJDDA, then New Jersey governor, Thomas Kean, vetoed the UDDA because it did not recognize any sort of accommodation for those who disagreed with DNC for religious reasons [4]. Many of the ideas on accommodating religious views that appear in the New Jersey law come from Robert Olick who notes, “the New Jersey Law…expresses a strong conviction that the societal need for uniformity should yield to and accommodate personal interests of a distinct minority of the population in the exercise of their religious beliefs. Meaningful protection demands empowerment of the individual through statutory recognition of a religious exemption” [5].

Beginning after the well-known Karen Quinlan case which occurred in New Jersey, vocal advocates called for greater attention to issues in medical ethics [6]. A New Jersey grassroots organization founded in 1983 called the Citizens’ Committee on Biomedical Ethics advocated for an official group to address medical ethics controversies [6]. Their efforts succeeded and in 1985, the New Jersey state government appropriated funding to create a permanent body called the New Jersey Commission on Legal and Ethical Problems in the Delivery of Healthcare, otherwise known as the New Jersey Bioethics Commission [4].

This Commission bought together experts representing a variety of interests and specialties in order to address healthcare ethics issues in the state through public hearings, commission meetings, and task forces [4, 6]. Its intent was both to encourage public discussion and create methods for governments to respond to unresolved biomedical ethics issues [7]. Before the creation of the Commission, public policy on biomedical controversies was typically formed after families pursued litigation [7]. The New Jersey Bioethics Commission was active for about six years, published its findings in six documents, and encouraged the creation of several state laws, such as the New Jersey Advance Directives for Health Care Act [8]. Just twenty-one years after its creation, it was formally eliminated due to inactivity and loss of funding [7, 9].

The Commission argued that although the definition of death should be rooted in scientific knowledge, the definition of death is actually a societal choice partially influenced by religion and so a religious exemption to DNC should be established [4]. It proposed the NJDDA before the NJDDA statute was passed and developed a guide for New Jersey residents to create end-of-life directives [8, 10].

In addition, Commission members hoped to pre-emptively resolve conflict about payment for medical treatment that occurred in *Cavagnaro v. Hanover Insurance Co.*, where a court ruled that life-support on a brain dead body being held for possible organ donation did not constitute medical treatment and held the patient’s family responsible for those expenses [4]. The New Jersey statute explicitly states, “No health care practitioner or other health care provider, and no health service plan, insurer, or governmental authority, shall deny coverage or exclude from the benefits of service any individual solely because of that individual’s personal religious beliefs regarding the application of neurological criteria for declaring death.”

There is, of course, some disagreement with the New Jersey Bioethics Commission’s support and establishment of religious exemptions to DNC. One argument is that any individual exceptions to the UDDA could undermine public policy by challenging the regular decisions to disconnect mechanical support for a brain dead patient, discouraging organ donations, preventing families from grieving a loss properly, and confusing the medical and legal boundary between life and death [11]. Others claim that many members of the public already have difficulty fully understanding DNC, especially since major media sources rarely define “brain death” or describe “determination of death” when discussing stories related to DNC and that religious exemptions would only compound this confusion [12].

Others point to the possibility of extended somatic survival and use of resources due to physiological support after neurological death. In a 1998 meta-analysis, Shewmon reported that he had found 175 cases of somatic survival after neurological death that lasted at least a week, 28 cases longer than a month, 17 cases longer than two months, and 4 cases longer than a year [13]. Since physiological support is often withdrawn, this data likely underestimates the current capabilities of medical technology to continue somatic survival [13]. The extended somatic survival of a brain dead body can be very unstable and require substantial resources and strategies to maintain [14, 15]. Physicians providing temporary physiological support to neurologically dead organ donors and pregnant women prior to organ donation and/or the development of the fetus reported that patients typically undergo a myriad of complications that can include tachycardia, hypothermia, diabetes insipidus, infection, deep vein thrombosis, pulmonary edema, and metabolic acidosis [14, 15].

Because of the controversies surrounding DNC, no national consensus exists on addressing medical definitions of determination of death while still preserving religious freedom. In addition to New Jersey, three states – California, New York, and Illinois – have statutes mandating that hospitals provide some religious accommodation for brain dead patients [16–18]. The religious accommodations in these states offer much less consideration for religious families involved in these disputes than New Jersey does. No comparable legislation exists in any other state at this time. Hospitals are still struggling to resolve the disputes between families and physicians when a patient’s family objects to DNC [19].

Objections to DNC are not unique to New Jersey, the only state where comprehensive religious exemptions exist. The ethics consultation service at Cleveland Clinic, located in a state where no legal religious exemption exists, reported thirteen requests for continued physiological support for a brain-dead patient between 2005 and 2013 [20]. Of these cases, three patient families cited reasons related to their faith in Orthodox Judaism, one referenced belief in God, and another named their Islamic faith [20]. Olick has raised the issue of whether it needs to be a specifically religious objection. In cases where persons identify as “non-religious” or “unknown”, it might be more appropriate to consider moral objections [21]. Many cases of religious objections to DNC in states without a religious exemption law may go unrecorded, unreported, ignored, or result in litigation.

Informed and ethical public policy to mediate this issue would help families who disagree with DNC because of their religious beliefs. Currently, when medical professionals or patients’ families turn to the courts, the decisions typically support the health care provider and rule in favor of discontinuing physiological support [22]. Although courts are capable of making decisions for specific conflicts, they are reactive, routinely find for the provider, and cannot preemptively write public policy to prevent disputes [7]. To avoid litigation and the additional distress it creates for families, there must be standardization at the state level and ultimately at the national level that accommodates both religious and moral objections to DNC. Standardization will also satisfy the current problem of families needing to change hospitals to obtain a more favorable environment for their religious and/or moral commitments.

Recent legal battles between families and health care providers on brain death have encouraged debates and greater consideration for both religious and moral exemptions from the public.^1^

Media outlets have reported several cases in which accepted medical standards of death conflicted with the religious dispensations of the patients’ families in states without religious exemption provisions.

Cho Fook Cheng, a brain-dead elderly Taiwanese Buddhist died at Beth Israel Deaconess Medical Center after a heart attack [24]. According to the religious beliefs of Cheng and his family, even if Cheng would never recover, removing the ventilator would eternally affect all his future lives as well as those of his family and health care providers [2]. Consequently, Cheng’s family filed a restraining order against the hospital which, in turn, contested the restraining order as an unreasonable request because Cheng’s body was physically deteriorating [24]. Eventually, Cheng’s family agreed to allow discontinuation of the intravenous medicine that kept his heart beating but were seriously disturbed by what they viewed as their loved one’s dishonorable and not peaceful death [24].

In 2008, a twelve-year-old boy, Motl Brody, lost his battle with brain cancer in Washington DC’s Children’s National Medical Center (CNMC) [25]. CNMC prepared to conduct a second, confirmatory test as mandated by protocol before declaring Brody brain dead [25]. However, Brody’s parents sought court intervention in order to block further testing or attempts to remove their son’s ventilator. The Brody family believed in a form of Orthodox Judaism that considers a beating heart and breath, even when mechanically supported, to be an indication of life [25]. CNMC argued that they needed the resources to provide medical treatment for other ill, living children [25]. Before the court rendered its decision, Brody’s body physically deteriorated until his heart stopped beating [26]. His case was never legally resolved [26].

Neither case had a final legal resolution. Had they occurred in New Jersey these court battles would not have been necessary. More information on the consequences of establishing such policy or recommending alternative approaches is needed to prevent similar cases in the future. There is not yet enough information on the true costs of religious exemptions to DNC, how frequently they occur, or the financial cost of litigation.

Considering the emotionally devastating impact of cases surrounding brain death and the complex factors raised by religious objections and continued physiological support, there is no simple solution to resolve such disputes. More knowledge on this subject may help policymakers consider all factors when forming new policy concerning religious objections to DNC.

The current lack of information on religious exemptions to DNC is unacceptable. Currently, the majority of states depend on courts that generally find for the provider.

The data collected on the frequency of religious exemptions to DNC will help provide clarification for the potential consequences of enacting such exemptions in more states. This information may help policymakers construct informed, ethical, and compassionate policy that both recognizes the struggles for religious freedom from families in such situations and reduces the need for litigation.

## Methods

New Jersey was selected for this study because of its provision for continued insurance coverage. This provision removes what might be a potential barrier to a family invoking the religious exemption clause of the NJDDA. This study was designed to obtain information on the frequency of religious exemptions to determination of neurological death in New Jersey. There is no other study that attempts to obtain this information.

To complete this study, literature and internet searches on neurological criteria of death, New Jersey bioethics organizations, advanced directives, public policy, and other related topics were conducted to increase understanding of the history and contemporary views on religious objections to DNC.

A list of hospitals and health networks was drawn from the New Jersey Hospital Association’s list of hospitals in the state [27]. Contact information for pastoral care (sometimes called spiritual care) departments or biomedical ethics committee heads was obtained through the websites of hospitals and health networks. Potential study participants were contacted by email (if possible) or by phone. They were invited to participate in a research interview and then asked about the frequency of religious exemptions in the past five years, what religious belief system (if any) was invoked, and whether they knew of any failed attempts at obtaining a religious exemption. Some participants elected to suggest additional people to contact for interviews.

All respondents were either chaplains or heads of hospital biomedical ethics committees currently working in New Jersey. Many of the chaplains were also members of a hospital bioethics committee. All had advanced degrees related to ministry or medicine and were at least eighteen years old.

Interviews were then conducted by phone from Boston at Northeastern University. Notes on these interviews were organized and compiled into the dataset reported in this paper.

## Results

Nineteen chaplains and bioethics committee heads, or 36% of the 53 contacted, agreed to participate in an interview. Of these, eighteen completed the interview; five reported an estimated 30–36 religious exemptions in the past five years. The three respondents that reported religious exemptions due to belief in ultra-orthodox Judaism worked at hospitals located in one of the top seven counties in New Jersey for Orthodox Judaism adherents and congregations [28].

## Discussion

Religious exemptions to DNC do occur in New Jersey, although infrequently (Table 1).^2^ The number of cases of religious exemptions reported by the respondents is very small. Considering the potential negative emotional impact on the religious groups who do disagree with DNC as well as the rarity of religious exemptions, it may be beneficial to grant religious exemptions to respect religious beliefs and reduce costly litigation, as the New Jersey Bioethics Commission originally intended.

**Table 1:** Results of Research Interview

Question	Response Options	Frequency	% of applicable responses
Was the respondent aware of religious exemptions?			
	Yes	15	79
	No	4	21
Was there a religious exemption at their hospital in the past five years?			
	Yes	5	28
	No	13	72
Approximate # of religious exemptions reported at the hospital in the past five years:			
	0	13	72
	1–5	2	11
	6–10	2	11
	11–15	1	6
Religious tradition of patient(s) obtaining religious exemption(s) at hospital:			
	Orthodox Jew	3	60
	Non-religious	1	20
	Unknown	1	20
Did the hospital deny any religious exemptions in the past 5 years?			
	Yes	0	0
	No	18	100

Surprisingly, roughly 21% of the respondents were unaware of the law that allowed for religious exemptions to DNC in New Jersey. The lack of statewide awareness of the religious exemption policy comes despite the New Jersey Bioethics Commission creation of a document intended to help members of the public draft advanced directives about their care [10]. The document explains religious exemptions to DNC as an option and is still recommended for use through the website of the New Jersey Department of Health [10].

All three hospitals where at least one religious exemption to DNC was reported are located in one of the top seven New Jersey counties for the greatest number of Orthodox Judaism adherents and congregations [28]. This could suggest that patients’ families and the rabbis they turn to for advice may be more aware of the availability of religious exemptions. In this study patient families from forms of Orthodox Judaism requested religious exemptions while patients from other traditions did not.

This study is likely underreporting the true number of religious exemptions to DNC in New Jersey. Religious exemptions to determination of neurological death were not officially tracked by hospitals and so gathering these data depends solely on the accurate recollection of willing study participants. A few participants had been working at the hospital for less than five years and so were unable to fully answer all the questions. Many acknowledged that they were only reporting the religious exemptions of which they were aware or had engaged professionally since there no records are maintained. This study depended heavily on personal experiences, many participants also used inexact terminology to report numbers.

Further research is necessary to determine the precise impact of religious exemptions to DNC. This is a report on religious exemptions in a limited number of hospitals; many of those contacted never responded to interview requests. Furthermore, this study did not ascertain the length of somatic survival of patients who obtained a religious exemption nor the potential negative effects of directing hospital resources to accommodate these religious exemptions. Without this information, there is no way to form a national consensus on how to address religious exemptions to DNC that both respects religious perspectives and considers the potential impact of granting religious exemptions. However, this does not eliminate the emotional effects a single religious exemption to DNC can have on a family with a strong faith in a religious tradition that objects to DNC. Religious freedom is a fundamental right in the United States. Exploring alternatives to the current policy of conflict, litigation, and ignoring religious beliefs would benefit future individuals personally affected by religious objections to DNC.

## Conclusions

Religious exemptions to DNC in New Jersey do occur, although they are rare. Prior to this study, there was no information on the frequency of religious exemptions. This study is most likely underreporting the total number of religious exemptions in the state; though the number is probably small. There is a need for either state or national policies that acknowledge religious objections to DNC. Allowing every individual case of religious objection to DNC to be resolved by courts is inefficient and costly. Furthermore, court rulings have not protected the religious freedom of patient families undergoing emotional ordeals when their loved one is declared dead by neurological criteria in a manner inconsistent with their religious beliefs.

More work is needed to better understand the impact of granting religious exemptions before new policy can be established; a system for tracking religious exemptions in New Jersey would provide important information. Medical technology will continue to develop, as will human capability to extend somatic survival after brain death. Ethical controversies surrounding brain death will continue to negatively affect patients and their families until they are resolved through consensus enforced by public policy. Although the New Jersey Bioethics Commission no longer exists, the importance of its original goals cannot be dismissed.

## Abbreviations

CNMC: Children’s National Medical Center
DNC: Death by neurological criteria
NJDDA: New Jersey Determination of Death Act
UDDA: Uniform Determination of Death Act

## Footnotes

1 Although it is not directly applicable to this research, there are a number of ongoing cases. The Jahi McMath case, which began in 2013, has brought much public attention to DNC and illustrates the difficulty that arises from differing state policies. Following surgical complications, McMath, was declared brain dead despite her family’s protests [23]. Following a legal battle, McMath was moved to New Jersey so that her family could request a religious exemption [23]. McMath’s family is contesting McMath’s status as brain dead rather than expressing religious objections to DNC [23]. Two ongoing Canadian cases, Shalom Ouanounou, an orthodox Jew and Taquisha McKitty, an apostolic pentacostal are both seeking religious exemptions from brain death determination. There are several ongoing cases in California, one of the more recent involving a toddler, Israel Stinson.

2 The total number of brain death donors for the state of New Jersey from 2012 to 2016 was 801, the period under study here [29].
